# Evaluating the Efficacy of Rib-to-pelvis Growth-friendly Surgery for the Treatment of Non-ambulatory Early-Onset Scoliosis Myelomeningocele Patients

**DOI:** 10.5435/JAAOSGlobal-D-22-00090

**Published:** 2022-05-06

**Authors:** Norman Ramirez, Gerardo Olivella, Ryan E. Fitzgerald, John T. Smith, Peter F. Sturm, Paul D. Sponseller, Lawrence I. Karlin, Scott J. Luhmann, Norberto J. Torres-Lugo, Tricia St. Hilaire, Pediatric Spine Study Group

**Affiliations:** From the Department of Orthopaedic Surgery, Mayagüez Medical Center, Mayagüez, PR (Dr. Ramirez); the Department of Orthopaedic Surgery, University of Puerto Rico, San Juan, PR (Olivella, and Dr. Torres-Lugo); the Department of Orthopaedic Surgery, Riley Children's Health, Indianapolis, IN (Dr. Fitzgerald); the Department of Orthopedics, Primary Children's Medical Center, Salt Lake City, UT (Dr. Smith); the Division of Orthopaedic Surgery, Cincinnati Children's Hospital, Cincinnati, OH (Dr. Sturm); the Department of Orthopaedic Surgery, Johns Hopkins University, Baltimore, MD (Dr. Sponseller); the Department of Orthopaedic Surgery, Boston Children's Hospital, Boston, MA (Dr. Karlin); the Department of Orthopaedic Surgery, Washington University School of Medicine, St. Louis, MO (Dr. Luhmann); and the Pediatric Spine Foundation, Valley Forge, PA (Dr. St. Hilaire).

## Abstract

Early-onset scoliosis (EOS) is a well-known orthopaedic manifestation in patients with myelomeningocele. The rib-based growing system (RBGS) has been proposed as an alternative for these individuals because of the poor outcomes with traditional surgical techniques. We aimed to describe the effect of RBGS in patients with nonambulatory EOS myelomeningocele.

**Methods:**

We retrospectively reviewed the Pediatric Spine Study Group Multicenter Database for all patients with nonambulatory EOS myelomeningocele treated with RBGS from 2004 to 2019. Demographics, surgical data, radiographic findings, and postoperative complications were obtained. The quality-of-life parameters were assessed postoperatively using the Early-onset Scoliosis Questionnaire-24.

**Results:**

Thirty patients (18 women; 60%) were patients with nonambulatory EOS myelomeningocele treated with RBGS. The mean age at the initial surgery was 5.3 years. The thoracic (T1-T12) spine height showed a significant increase from initial surgery to the most recent follow-up (*P* < 0.001). Spine (T1-S1) height was also significantly increased (*P* < 0.001). The postoperative complication rate was 87%. The Early-onset Scoliosis Questionnaire-24 demonstrates significant improvements in the quality-of-life scores (*P* = 0.037).

**Conclusion:**

This study demonstrated that RBGS could improve the reported quality-of-life scores in patients with nonambulatory EOS myelomeningocele when assessed with an EOS-oriented tool. Moreover, we confirmed the ability of RBGS to hold or even correct spinal deformity.

Myelomeningocele is a saclike structure containing cerebrospinal fluid and neural tissue that occurs in approximately one in 1,000 live births due to a failure of the neural tube to close during the fourth week of gestation.^1^ Throughout history, scoliosis has been reported as it is the most common orthopaedic manifestation, with an overall incidence of 50% to 80% in patients with myelomeningocele.^2,3,4,5,6,7^ In this subset of patients, scoliosis is usually characterized by an early onset and rapid progression.^3,7^ The pathogenesis of spinal deformity in patients with myelomeningocele is multifactorial, including congenital vertebral anomalies, hydrocephalus, shunt malfunction, Chiari malformations, tethered cord, or hydromyelia.^3,5-7^ The manifestation of scoliosis is highly correlated with the level of the last intact laminar arch/spinal dysraphism and the clinical level of paralysis.^3,6-8^ In the same way, scoliosis development inversely correlates with ambulatory abilities, with high rates of scoliosis seen in nonambulatory patients with poor trunk control.^3,5,6,7,8,9^

It is well known that the spinal deformity in this population represents a notable burden for patients and their caregivers.^7,9,10^ This abnormality leads to a loss of spine height, causing difficulties in sitting, eating, and urination because of an increase in the chest and abdominal pressures.^10,11^ In addition, patients could experience breathing difficulty because of reduced chest wall compliance and uneven lung expansion producing an ineffective length-tension relationship of the respiratory muscles with a subsequent decline in pulmonary function.^12^ Consequently, these patients are more prone to develop progressive severe restrictive pulmonary disease, nocturnal/diurnal respiratory failure, ventilator dependence, and early death.^11,12,13,14^

Currently, the primary treatment target has been centered on limiting spinal curve progression, improving respiratory system development, preventing bedsores, and releasing the upper limbs from the duty of supporting the trunk.^3,5,9^ However, in patients with early-onset scoliosis (EOS), fusion in situ and hemiepiphysiodesis have failed to manage the thoracic deformity, restricting spine and lung growth.^9,15^ In 2003, Campbell et al^16^ developed the rib-based growing system (RBGS), a novel surgical approach to control spinal deformity, allowing vertebral column growth and lung parenchymal development in EOS. Owing to the poor surgical outcomes of spine fusion, RBGS was recommended as a treatment alternative to EOS in patients with nonambulatory myelomeningocele.^9,10,15-17^ However, the long-term surgical outcomes in this group of patients remain limited.

The aim of this study was to describe the radiographic parameters, health-related quality-of-life outcomes, and complications of patients with nonambulatory EOS myelomeningocele treated with a double rib-to-pelvis RBGS as treatment of their progressive spine deformity.

## Methods

We conducted a retrospective review from the Pediatric Spine Study Group (PSSG) multicenter EOS database of all patients with nonambulatory myelomeningocele EOS treated with double rib-to-pelvis RBGS from 2004 to 2019. Inclusion criteria were set for nonambulatory skeletally immature myelomeningocele patients with progressive spinal deformity, no previous treatment, a minimum follow-up period of 2 years, and an RBGS with three or more lengthening procedures. Seven pediatric spine centers in the United States and Puerto Rico participate in this project under Institutional Review Board approval.

The indication for the RBGS was the evidence of progressive scoliosis secondary to a congenital or neurogenic source.^9,17^ This surgical technique involved three incisions to insert implants on both sides of the midline, where the upper cradle was attached from the second to fourth ribs and distally to the pelvis with an iliac hook. The locking mechanism was inserted and connected through a subfascial tunnel on both sides of the spine, as described by Smith.^9,15^ As a standardized protocol, the lengthening procedures were done every 6 months.

Demographics, surgical data, radiographic findings, quality-of-life parameters, and postoperative complications were evaluated. Demographic data included sex, age, body mass index (BMI), comorbidities, and follow-up time. Surgical data encompassed index procedure time and length of hospital stay, estimated blood loss, and the number of lengthening procedures.

Radiographic data included the preoperative, immediately after surgery, and last follow-up coronal and sagittal Cobb angle, thoracic spine height (T1-T12 height), and thoracolumbar spine height (T1-S1 height). Radiographic measurements for the T1-T12 height were taken from the center of the T1 superior end plate to the center of the T12 inferior end plate while the T1-S1 height was taken from the center of the T1 superior end plate to the center of the S1 superior end plate.^9,14^

The quality-of-life parameters were evaluated using the Early-onset Scoliosis Questionnaire (EOSQ-24). Patients' parents or legal guardians completed this questionnaire to assess the disease's quality of life and burden through 24 items. The validated tool consists of eight health-related quality-of-life (HRQOL) domains and four domains related to the effect on parents and satisfaction of the child and parents.^18-20^ Each item was answered on a scale from 1 (not relevant) to 5 (very relevant).^19^ The score of each item was calculated as follows: (average value of item choice − 1)/4×100. The mean of all 24 items is the total quality-of-life score, with a potential range from 0 (poor) to 100 (excellent).^19^

Postoperative complications were defined as any change from the normal postoperative course that occurred from the time of surgery until the most recent follow-up visit. The complications were illustrated by rate, type, and severity as described in previous studies.^21^ The severity grade of postoperative complications was reported using a standardized system based on the severity and effect of the surgical treatment, as described by Smith et al.^21,22^

The characteristics of the sample data were described using mean, SD, and ranges for continuous variables and frequency and percentage for categorical variables. Comparisons between the values before the index surgical intervention, immediately after surgery, and the most recent follow-up were done using the paired *t*-test. The SPSS and Microsoft Excel software were used to perform the statistical analysis. A *P*-value of <0.05 was considered statistically significant.

## Results

From all patients enrolled in the PSSG database, 30 cases met the inclusion criteria. Forty percent of them were male (12 patients), and the mean age for initial placement of the RBGS was 5.3 ± 2.6 years (range: 2.7 to 9.0 years). Weight increased from 18.4 ± 5.5 kg (range: 10.4 to 33.3) to 34.8 ± 12.5 kg (range: 19.5 to 67.1) at the most recent follow-up. The mean BMI before surgery was 18.1 ± 2.9 kg/cm^2^ (range: 13.2 to 23.7 kg/cm^2^), and the most recent BMI was 21.2 ± 8.4 kg/cm^2^ (range: 15.2 to 41.6 kg/cm^2^). The mean length of follow-up between the index surgery and the latest available follow-up was 4.9 ± 2.1 years (range: 2.8 to 9.0 years).

These patients presented with multiple associated medical conditions such as hydrocephalus with a ventricular peritoneal shunt in all patients, chronic urinary tract infection in 20 patients, respiratory problems (ie, sleep apnea and/or fatigue; no requirement of ventilatory assistance and/or tracheostomy) in seven patients, rib fusions in four patients, chronic skin ulcers in four patients, and cardiac problems in three patients.

**Figure 1:**
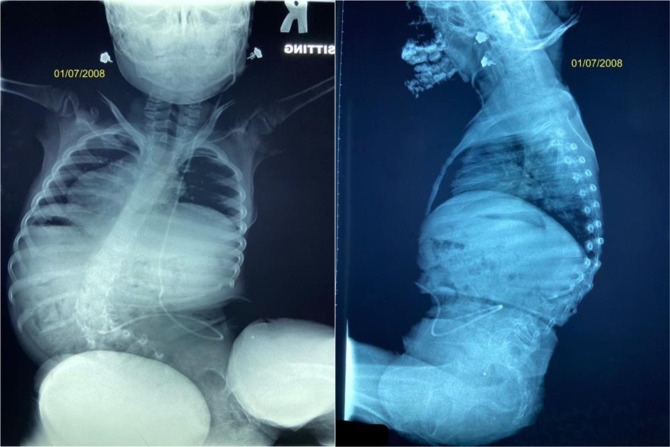
Radiograph showing a six-year-old female patient with a thoracic level nonambulatory myelomeningocele.

**Figure 2:**
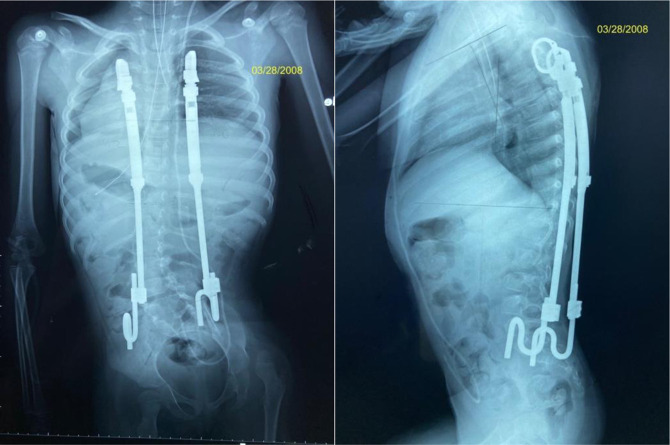
Radiography showing the status post bilateral rib-to-pelvis growing system.

The surgical outcomes demonstrated an index procedure mean length of hospital stay of 4.2 ± 2.0 days (range 2.0 to 9.0 days), and the mean initial surgery procedure time was 194.0 ± 90.9 minutes (69.0 to 503.0 minutes). The mean estimated blood loss of the implantation was 59.7 ± 39.4 mL (range: 30 to 150 mL). The mean number of lengthening procedures was 6.6 ± 2.9 (range: 3.0 to 14.0). See Figures 1 and 2.

Before implantation, the mean primary Cobb angle was 67.8 ± 30.5° (range 35 to 111.0) and the immediate post-RBGS implantation mean Cobb angle significantly decreased to 41.1 ± 20.1° (range: 9.0 to 79.0) (*P* < 0.0001). However, this change in the Cobb angle decreased with time, with a final average Cobb angle reaching 60.9 ± 27.6° (range: 10.0 to 104.0). The mean initial thoracic kyphosis was 39.1 ± 25.8° (range: 2.0 to 111.0), the immediate post-RBGS thoracic kyphosis was 30.4 ± 13.1° (range: 6.0 to 56), and the last thoracic kyphosis angle (T2-T12) was 31.7 ± 17.3° (range: 1.0 to 69.0).

During the treatment period, all 30 patients experienced an increase in T1-T12 height, with an average change of +5.0 cm. A significant average change was noted from the starting thoracic (T1-T12) spine height (15.3 ± 3.6 cm [range: 10.27 to 25.11]) to the last evaluation (22.2 ± 4.8 cm [range: 18.9 to 27.8]) (*P* < 0.001). Spine height (T1-S1) significantly increased from a mean of 22.1 ± 6.2 cm (range: 10.3 to 37.9) pretreatment to 35.3 ± 7.9 cm (range: 29.9 to 44.3) at the last follow-up evaluation (*P* < 0.001).

The EOSQ-24 was introduced in the PSSG database in 2012; therefore, all the EOSQ-24 scores of the patients that meet our inclusion criteria were obtained after the index procedure. A total of 16 patients had three or more fully completed EOSQ-24 scores after the index surgery with at least 6 months apart. The mean initial total EOSQ score was 62.3 (range: 36.9 to 81.5), and the last total EOSQ score was 69.4 (range: 44.6 to 91.7) of a possible 100 points, which was statistically significant (*P* = 0.037). An improvement was found in all EOSQ-24 domains; however, only in General Health and Parents Satisfaction Domains, these changes were statistically significant (General Health *P* = 0.004, Parents Satisfaction *P* = 0.047) (Table 1).

**Table 1:** Comparison Between Preindex Surgery and Most-recent Follow-up Quality-of-life Scores

Score Categories	Preindex, Mean ± SD (Range)	Most Recent Follow-up, Mean ± SD (Range)	*P*
General health	55.77 ± 14.0 (37.5-87.5)	73.08 ± 16.01 (37.5-100)	0.00365
Pain/discomfort	75.96 ± 16.51 (62.5-100)	85.58 ± 14.29 (62.5-100)	0.12687
Pulmonary function	82.69 ± 22.56 (37.5-100)	91.35 ± 12.89 (62.5-100)	0.14500
Transfer	76.92 ± 29.69 (25.0-100)	69.23 ± 25.32 (25.0-100)	0.45494
Physical function	49.36 ± 33.59 (0.00-91.67)	66.03 ± 33.54 (0.00-100)	0.13247
Daily living	37.50 ± 26.02 (0.00-87.5)	45.19 ± 32.49 (0.00-100)	0.34478
Fatigue/energy level	66.35 ± 22.47 (37.5-100)	67.31 ± 23.13 (37.5-100)	0.89712
Emotion	72.12 ± 26.59 (25.0-100)	75.96 ± 23.64 (25.0-100)	0.59950
Parental impact	61.54 ± 21.54 (25.0-100)	60.00 ± 17.44 (30.0-100)	0.76840
Financial impact	65.38 ± 36.14 (0.00-100)	73.08 ± 27.88 (25.0-100)	0.51472
Parents satisfaction	56.73 ± 26.82 (25.0-100)	72.12 ± 20.51 (37.5-100)	0.04713
Total	62.27 ± 14.15 (36.96-81.5)	69.38 ± 13.74 (44.6-91.7)	0.03709

Postoperatively, there were a total of 61 complications reported, with a complication rate of 86.6% (26/30 patients). Device rod migration and related issues (50.8% = 31/61 complications) were the most common postoperative complications, followed by implant failure (26.2% = 16/61 complications), infection and wound dehiscence (16.4% = 10 of 61 complications), pain (3.3% = 2/61 complications), respiratory distress (1.6% = 1/61 complications), and one death related to patients' natural disease (1.6% = 1/61 complications). Device migration was defined as complete implant displacement to the lateral aspect of the iliac crest or medially to the sacroiliac joint. No intraoperative deaths were reported. The most common severity grade reported was II-B (requiring multiple unplanned surgical events). The severity grade and rate of postoperative complications per patient are presented in Table 2.

**Table 2:** Postoperative Complications and Severity Grade

Complications	Frequency (%)
Severity grades (SVs) for complications	
SV–I: Corrected at planned surgery	7 (26.9)
SV–II-A: Single unplanned surgical event	7 (26.9)
SV–II-B: Multiple surgical events	10 (38.5)
SV–III: Permanent abandonment of surgical technique and device removal	1 (3.8)
SV–IV: Death during treatment	1 (3.8)
Rate of complication per patient	
Patient with no complications	4 (13.3)
Patient with one complication	12 (40.0)
Patient with two complications	2 (6.7)
Patients with three complications	5 (16.7)
Patients with four complications	2 (6.7)
Patients with five complications	4 (13.3)
Patients with six or more complications	1 (3.3)
Type of complication	
Device migration and related issues	31 (50.8)
Implant failure	16 (26.2)
Infection and wound dehiscence	10 (16.4)
Pain	2 (3.3)
Death	1 (1.6)
Pneumothorax	1 (1.6)

Fifty of the 61 postoperative complications (50/61 = 82.0%) were resolved by surgical intervention. Among the device migration and related issues (26/31 = 84%), complications required surgical implant repositioning. All implant failure-related complications (16/16 = 100%) required an implant exchange. Half of the postoperative infections (5/10 = 50% complications) were resolved with antibiotics while the other half (5/10 = 50% complications) underwent cleansing and débridement without implant removal but remained on suppressive antibiotics. Finally, neurogenic pain and respiratory distress complications were resolved with medical intervention.

## Discussion

The natural history of untreated nonambulatory myelomeningocele patients with severe EOS and their treatment alternatives have been an area of growing investigation.^7,10,15,17,18^ Achieving deformity correction, promoting good spinal and lung development, imposes a treatment challenge. The traditional surgical options for deformity correction, such as fusion or epiphysiodesis, usually lead to a short trunk, crankshaft deformity, spine and lung growth inhibition, or premature death.^9,15^ Considering the adverse effects of the traditional surgical options, the rib-based growing technique was developed and presented in 2003 as a surgical alternative to address the complex surgical pathology in this group of patients.^16,19^ The RBGS provides the mechanical ability to control spinal deformity and allow for continuous vertebral column lengthening and lung parenchymal growth.^9,15,17,19^ Our data confirmed that the bilateral rib-to-pelvis rib-based growing technique is an adequate procedure for EOS management in nonambulatory myelomeningocele patients with satisfactory radiographic and quality-of-life outcomes, although with a high complication rate considering the challenging nature of these patients.

At this moment, there are two published studies focused on the surgical outcomes of patients with nonambulatory myelomeningocele EOS treated with the RBGS.^9,17^ In 2011, Flynn et al published the earliest series of uniformly surgically treated spinal deformities in patients with EOS in patients with nonambulatory myelomeningocele treated with an RBGS insertion followed by a regular lengthening. They found the RBGS an adequate option to maintain or improve spine deformity in this group of patients. However, they recognized the limited number of patients (16 patients) and the short follow-up as notable study limitations.^17^ In 2014, Abol and Stuecker recommended this technique (ie, RBGS) as the preferred method of instrumentation in myelodysplastic patients because it allows a fixation lateral to the scar of the midline incision, away from the absent or abnormal posterior elements of the myelodysplastic spine, and away from the occasional local kyphosis observed in the midline, which potentially decreases the risk of infection and increases the corrective moment arm.^9,15^ Yet, their study suffers the same patient limitation (7 patients), restraining the power of their conclusions.^9^ Our study overcame the limitation of previous studies, collecting the patients (30 patients) through a multicenter registry with a longer follow-up (5 years) despite the decreasing incidence (1.9/10,000 births) of spina bifida in recent years because of the addition of folate in the diet of women of childbearing age, hindering the recruitment of patients with myelomeningocele.^7,23^ We retrieved the data for all the patients that meet our inclusion criteria with only two individuals previously included in the study by Smith et al.^15^

The rate of spinal deformity progression in patients with nonambulatory spina bifida is unclear because of the lack of long-term natural history studies of untreated patients.^7,17^ However, an increase in spinal deformity, loss of the spine alignment, and functional impairment with subsequent deterioration of respiratory function could be anticipated.^7,17^ In 1984, Muller et al developed a multivariate model to predict the curve progression in myelodysplastic patients. The model involved several variables such as the deformity angle, ambulatory potential, functional level, and age. In those patients with a curve more than 40° at initial evaluation, nonambulatory with a high lumbar or thoracic functional level, and less than 10 years, the estimated progression rate was calculated at 13° per year.^6^ After the Muller multivariate model, in a group of patients with an initial Cobb angle of 68° followed by 5 years without treatment, the expected Cobb angle should be more than 130°.^6^ However, after the rib-based growing rod treatment and scheduled lengthening, the most recent average Cobb angle was 61°, exposing the treatment potential to hold deformity progression.

A notable reduction in Cobb angle correction after repeated lengthening was observed in this group of patients. The initial mean primary Cobb angle was 67.8°, which decreased to 41.1° immediately after the index procedure. However, this change in the Cobb angle decreased with time, with a final average Cobb angle reaching 60.9°. These changes go in concordance with those observed in a previous standard growth rod system study.^17^ The loss of correction has been associated with the combination of stiffness from the soft tissues around the spine and the fragility of bone due to the lack of mobility, weight bearing, and nutritional deficiencies.^9,17^

The thoracic spinal growth prediction in the spina bifida population has not been defined.^17^ In a normal population, DiMeglio and Bonnel reported a thoracic spinal growth of 1.4 cm per year during the first 5 years and 0.6 cm per year from age 5 to 10 years.^24^ In our series, the mean thoracic spinal height (growth) by year after the initial procedure was 1.0 cm by year, which validated one of the surgical goals of this novel surgery. In addition, all the patients reached the critical height (T1-T12) more than 18 cm, as recommended by Karol et al.^25^

How the surgical spine procedures in nonambulatory myelodysplastic patients affect the quality of life have been poorly weighed. Previous studies focused on skeletally mature patients with spina bifida have questioned the benefits of posterior spine fusion in this group of patients.^26,27^ Most of the studies used several tools, such as Activities Scale for Kids, Quality of Life in Spina Bifida Questionnaire, the Pediatric Outcomes Data Collection Questionnaires, or Spina Bifida Spine Questionnaire, to evaluate the quality-of-life issues. However, these instruments were not oriented to evaluate the EOS population.^28,29^ The EOSQ-24 is the first disease-specific, parent-reported HRQOL outcome measurement tool for this condition.^18-20^ This instrument has been used to evaluate the quality of life in EOS since its creation.^18-20^ Up to this date, all the studies have found that the neuromuscular EOS group scored markedly lower general EOS-Q scores than those with congenital, idiopathic, or syndromic EOS categories.^18,19^ None of the previous studies regarding the use of RBGS in nonambulatory myelodysplastic patients had addressed the effect of the surgical procedure in the HRQOL parameters.^9,10,17^ The success of the scoliosis treatment had been measured only through radiological parameters such as reduction of spinal deformity, spine height changes, pelvic tilt correction, and increased space availability for lung expansion with an evaluation of the complication rate in this complex population.^9,10,17,19^ As the deformity progresses, it is expected that the overall quality-of-life parameters of these patients will deteriorate.^18,19^ “None of the patients complained of functional impairment from the instrumentation, the majority having reported a subjective sensation of improved sitting stability and better freedom of the hands for uses other than trunk support” was the only comment regarding the quality of life in the study by Aboul Oyoun et al.^9^ Our study, contrary to the negative outcomes of posterior spine fusion in patients with skeletally mature spina bifida, demonstrates that the RBGS improves the quality-of-life scores in nonambulatory myelomeningocele patients with EOS when assessed with an EOS-oriented tool.

The postoperative complication rate in nonambulatory myelodysplastic patients has been a persistent concern with all surgical alternatives.^5,7,9,10,17^ Usually, they suffer from different comorbidities, such as hypoventilation syndrome, pulmonary infections, intellectual disability, urinary infections, epilepsy, Arnold Chiari herniation, nutritional compromise, and bowel incontinence, increasing their predisposition to complications. Moreover, the rib-based growing instrumentation has many potential complications, well described in the literature.^9,10,17^

Complications are expected when a challenging group of patients is treated with a high morbidity procedure. However, the benefits of the surgical intervention (hold curvature progression, increased spine height, and improved quality-of-life parameters) exceed the severity of the potential complications. In our study, the most common complication was implant migration and failure, which has been previously reported.^10,17^ Forty percent of the patients sustained only one complication, but most of the time required multiple unplanned surgical interventions. The high complications rate (87%) reported in this study is comparable with the rates seen in previous studies of RBGS in patients with nonambulatory neuromuscular EOS.^9,17,30^

Our study has some limitations. First, the study's retrospective design represents constraints in information availability (ie, EOSQ-24 scores were available only after the index procedure) and introduces some bias in patients' selection. Second, the incidence of myelomeningocele has decreased because of folate supplementation as part of prenatal care, limiting patient recruitment and resulting in a small sample size. Third, the EOSQ-24, despite being a validated tool, does not have a minimal clinically important difference established, providing a certain restriction to the clinical significance of our reported scores. Finally, using a questionnaire as a research tool represents disadvantages such as differences in question interpretations, uncertain answers, or unconscientious responses.

## Conclusion

This study represents the first assessment on the effect of using the RBGS in the health-related quality-of-life parameters in nonambulatory myelodysplastic patients. Our results demonstrate that the RBGS improves all the health-related quality-of-life scores when adequately assessed through an EOS-oriented tool. Despite the high risk of complications associated with this procedure, the primary goals of surgical management (ie, hold deformity progression, increase spine height, and improve quality of life) were achieved based on the study outcomes. These findings provide valuable information to orthopaedic surgeons who manage nonambulatory myelodysplastic patients when discussing the treatment alternatives and their potential benefits in outcomes and quality of life.
